# Comparative analysis of the complete mitochondrial genome sequences and anther development cytology between maintainer and Ogura-type cytoplasm male-sterile cabbage (*B. oleracea* Var. *capitata*)

**DOI:** 10.1186/s12864-021-07963-x

**Published:** 2021-09-07

**Authors:** Xionghui Zhong, Denghui Chen, Jian Cui, Hailong Li, Yuxin Huang, Jungen Kang

**Affiliations:** 1 Beijing Vegetable Research Center, Beijing Academy of Agriculture and Forestry Sciences, Key Laboratory of Biology and Genetic Improvement of Horticultural Crops (North China), Ministry of Agriculture, 100097 Beijing, P.R. China; 2 College of Horticulture, Gansu Agricultural University, 730070 Lanzhou, P.R. China

**Keywords:** Cytoplasmic male sterility (CMS), *orf138a*, *orf154a*, Mitochondrial genome, Tapetal cell

## Abstract

**Background:**

Cytoplasmic male sterility (CMS) has been widely used for commercial F1 hybrid seeds production. CMS is primarily caused by chimeric genes in mitochondrial genomes. However, which specific stages of anther development in cabbage are affected by the chimeric genes remain unclear.

**Results:**

In the present study, the complete mitochondrial genomes were sequenced and assembled for the maintainer and Ogura CMS cabbage lines. The genome size of the maintainer and Ogura CMS cabbage are 219,962 bp and 236,648 bp, respectively. There are 67 and 69 unknown function ORFs identified in the maintainer and Ogura CMS cabbage mitochondrial genomes, respectively. Four *orfs*, *orf102a*, *orf*122b, *orf*138a and *orf*154a were specifically identified in the Ogura CMS mitochondrial genome, which were likely generated by recombination with Ogura type radish during breeding process. Among them, ORF138a and ORF154a possessed a transmembrane structure, and *orf138a* was co-transcribed with the atp8 and *trnfM* genes. *orf154a* is partially homologous to the ATP synthase subunit 1 (*atpA*) gene. Both these genes were likely responsible for the CMS phenotype. In addition, cytological sections showed that the abnormal proliferation of tapetal cells might be the immediate cause of cytoplasmic male-sterility in Ogura CMS cabbage lines. RNA-seq results showed that *orf138a* and *orf154a* in Ogura CMS might influence transcript levels of genes in energy metabolic pathways.

**Conclusions:**

The presence of *orf138a* and *orf154a* lead to increased of ATPase activity and ATP content by affecting the transcript levels of genes in energy metabolic pathways, which could provide more energy for the abnormal proliferation of tapetal cells. Our data provides new insights into cytoplasmic male-sterility from whole mitochondrial genomes, cytology of anther development and transcriptome data.

**Supplementary Information:**

The online version contains supplementary material available at 10.1186/s12864-021-07963-x.

## Background

Plant male sterility refers to the failed production of normal anthers, pollen and male gametes. Male sterility is an efficient and cost-effective way to utilize heterosis in F1 seed production in many crops, such as corn, rice, wheat, and some species of brassica vegetables. Male sterility is classified into cytoplasmic male sterility (CMS) and genic male sterility (GMS) according to its genetic basis. CMS is caused by the interaction between the cytoplasm and nuclear genes [1]. However, GMS is controlled by nuclear genes alone [2].

The CMS of *Brassica oleracea* derives from sterile cytoplasm of other cruciferous species, including *B. rapa*, *B. nigra*, *B. napus* and *Rahanus sativus*. The *ogu* CMS, *nap* CMS, *pol* CMS, and *hau* CMS systems have been studied widely and used practically in *Brassica* breeding. Ogura CMS was discovered from a wild variety of Japanese radish (*R. sativus*) [3]. This radish CMS was first transferred into *B. oleracea* through distant hybridization and consecutive backcrossing in 1974 [4]. *nap* CMS was first found in rapeseed in 1971, and was used for heterosis breeding in 1973 [5, 6]. Polima (*pol*) CMS of *B. napus* is another well-studied male sterility type, which was transferred into *B. oleracea* by protoplast fusion [7]. *hau* CMS was first identified in *B. juncea*, and the male sterility was transferred to *B. napus* by interspecific hybridization [8]. With the development of DNA sequencing technology, more and more mitochondrial and chloroplast genomes of CMS lines have been sequenced completely. Consequently, comparative genomics showed that CMS is caused by rearrangement of the mitochondrial genome. Furthermore, some novel chimeric open reading frames (ORFs) generated by mitochondrial recombination have been reported as the determinants of CMS.

To date, the CMS-specific ORFs could be categorized into three groups : (1) ORFs that are co-transcribed with the adjacent upstream or downstream functional genes ; (2) ORFs located in the unique region of the mitochondrial genome of sterile lines, which are mostly similar to mitochondrial sequences of other species or have no homologous sequence in the known databases; and (3) ORFs possessing a chimeric structure, which have patial homologous sequences to known genes. For example, *orf138* has been considered as the determinant of Ogura CMS in *Brassiceae* [9]. Usually, *orf138* is found to be co-transcribed with *atp8* and *trnfM* [10]. In *B. napus*, *orf222* is regarded as the master control genes for *nap* male-sterility. In addition, *orf224* is responsible for *pol* male-sterile of *B.napus* and *orf224* is co-transcribed with atp6 [1]. Co-expression of *orf222* with *nad5c* and *orf139* might be the cause of *nap* CMS [11]. The chimeric CMS-associated gene *orf288* is a CMS-associated gene in the *hau* CMS line of *B. juncea*. Moreover, *orf288* possess a partial sequence of *nad5* and is co-transcribed with the downstream gene *atp6* [12, 13]. The different CMS-associated genes all cause similar phenotypic male sterility in different plants. It is generally agreed that anther-specific gene expression would represent straightforward evidence for CMS; however, none of CMS-associated genes show anther-specific expression, therefore, the interaction of CMS-associated genes with the anther development pathways requires further study.

Therefore, four hypothetical models have been proposed to demonstrate the mechanism of CMS: the cytotoxicity model, the mitochondrial energy deficiency model, the aberrant tapetal programmed cell death (PCD) model, and the retrograde regulation model [14]. For the CMS protein cytotoxicity model, ORF138 and ORF288 proteins have been found to be toxic to *E.coli*; however, there is lack of direct cytotoxicity evidence in plant anther cells [13, 15, 16]. Mitochondria, as the cell “energy factories”, supply ATP to meet the substantial energy requirements of anther development; disorders of mitochondrial functions could have dramatic effects on energy production, which will trigger male sterility. Many studies have reported that some CMS-associated genes are co-transcribed with subunits of the electron transport chain and ATP synthase complexes [1, 10]. This characteristic strongly suggests a link between the energy deficiency hypothesis and CMS. In the aberrant programmed cell death model, tapetum functions in surrounding and nurturing the pollen grains [17] and abnormal (advanced or delayed) tapetal PCD results in the CMS [18–20]. The hypothesis of the retrograde regulation model is based on alteration in nuclear gene expression as a result of signaling from mitochondria to the nucleus, which is induced with genetically or metabolically [21]. For instance, carrot ‘carpeloid’ CMS is caused by mitochondrial influence on the expression two MADS box genes [22]. Furthermore, the expression level of ORF11 under rice CW cytoplasm determines fertility or CMS, which was also explained using the retrograde signaling model [23].

In the present study, through comparative analysis of the maintainer and Ogura CMS sequenced mitochondrial genomes, we found that the cytoplasm of Ogura CMS was likely generated by recombination with Ogura-type radish during the breeding process. Four *orfs* (*orf102a*, *orf122b*, *orf138a* and *orf154a*) from radish were specifically identified in the Ogura CMS mitochondrial genome. *orf138a* was co-transcribed with atp8 and *trnfM*. Interestingly, ORF154a also possessed a transmembrane structure and its gene is located between Ogura CMS block 4 and Unique region II. However, which stage of anther development is affected by these chimeric genes remains elusive in cabbage. Our cytological sections data showed that the abnormal proliferation of tapetal cells might be the immediate cause of cytoplasmic male-sterility in Ogura CMS cabbage line. RNA sequencing (RNA-seq) results indicated that the transcript levels of genes in energy metabolic pathways in Ogura CMS were affected by mitochondrial recombination. Finally, our data provides new insights into the CMS process through comparative analysis of whole mitochondrial genomes, the cytology of anther development, and transcriptome data.

## Results

### Assembly of mitochondrial genome sequences of maintainer and Ogura CMS lines

**Fig. 1:**
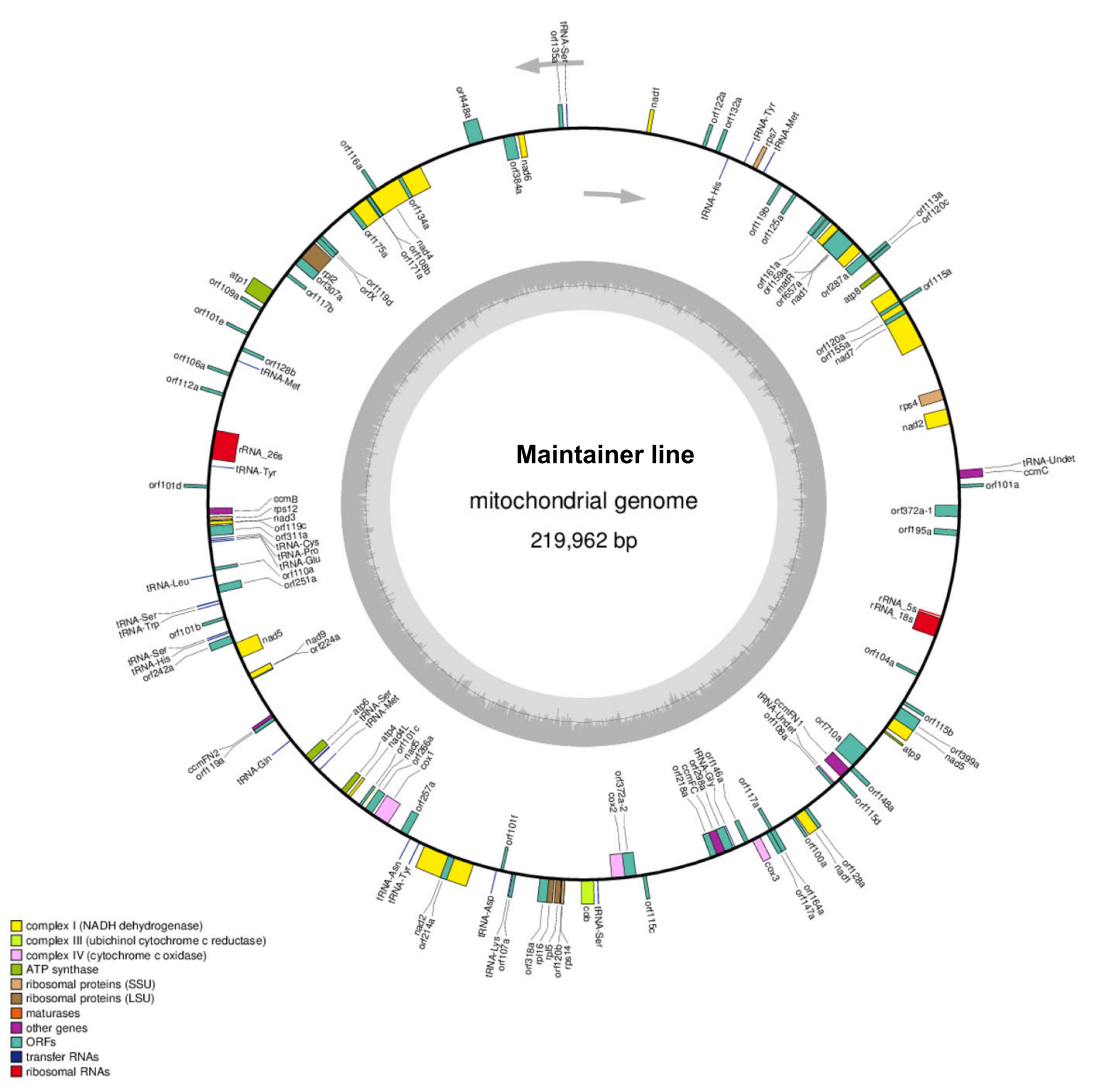
The mitochondrial genome map of the maintainer line. Genes with names inside the circle are transcribed clockwise. Genes with names outside the circle are transcribed counterclockwise. The colors of the genes denote the functions of the gene products

**Fig. 2:**
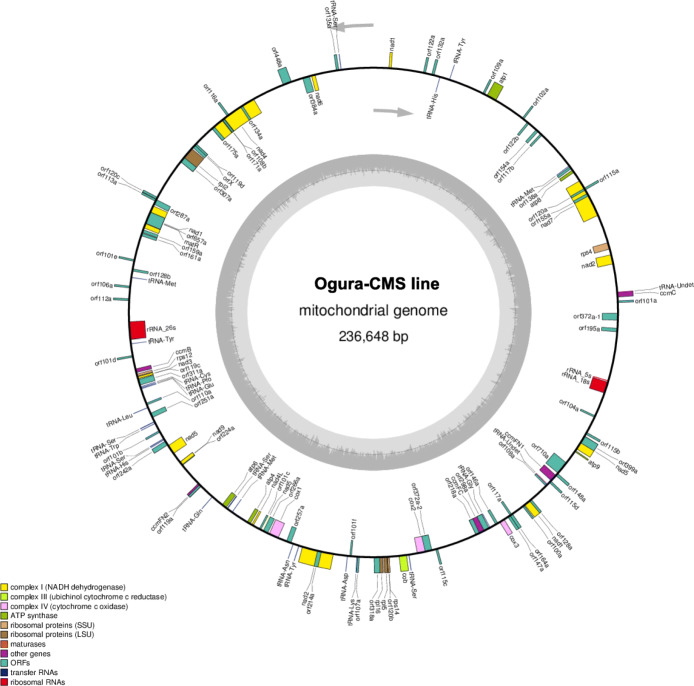
The mitochondrial genome map of the Ogura CMS line. Genes with names inside the circle are transcribed clockwise. Genes with names outside the circle are transcribed counterclockwise. The colors of the genes denote the functions of the gene products

The mitochondrial genomes of the maintainer and Ogura CMS cabbage lines were sequenced using Illumina Hiseq and PacBio Sequel techniques. In the Illumina Hiseq data, 8399 Mb and 9036 Mb of raw data were generated from the maintainer and Ogura CMS lines, respectively. We obtained 4983 Mb and 6197 Mb of clean data after filtering out adapters, reads containing over 10 % Ns and low-quality reads, respectively. The average of the Phred scores (Q20 and Q30) were calculated as over 94.71 and 96.81 % for these two cabbage samples. In the PacBio Sequel data, 96.37 Mb and 196.47 Mb subread bases were produced, the average length of the subreads were 8667 and 8684 bp after filtering the low-quality polymerase reads in the samples of maintainer and Ogura CMS and cabbage lines, respectively. The mitochondrial genomes of the maintainer and Ogura CMS cabbage lines were assembled into a single, circular molecule with sizes of 219,962 bp and 236,648 bp, respectively. The GC contents of mitochondrial genomes were 45.26 and 45.38 %, respectively (Figs. 1 and 2). The complete mitochondrial genome sequences of the maintainer and Ogura CMS lines were deposited in the GenBank nucleotide sequence database (https://www.ncbi.nlm.nih.gov/genbank/) under the accession numbers MW423604 and MW423605, respectively.

### Comparative analysis of protein-coding genes in the mitochondrial genomes

We identified 31 known genes, 67 ORFs, 3 rRNA genes (5 S, 18 and 26 S), and 25 tRNA genes in the maintainer line genome using BLAST and tRNA-SE (Supplementary Table 1). Mitochondria are known as the cell “powerhouse”, which supply ATP through oxidative phosphorylation complexes. The 31 known genes are involved the electron transport chain and ATP synthase, such as nine subunits of complex I (*nad1*, *2*, *3*, *4*, *4 L*, *5*, *6*, *7*, and *9*), one subunit of complex II (*cob*), three subunits of complex IV (*cox1*, *2* and *3*) and five subunits of complex V (*atp1*, *4*, *6*, *8*, and *9*). In addition, there were five genes (*ccmB*, *ccmC*, *ccmFN1*, *ccmFN2* and *ccmFC*) that participate in cytochrome c biogenesis, seven ribosomal genes (*rpl2*, *rpl5*, *rpl16*, *rps4*, *rps7*, *rps12* and *rps14*) and one maturase gene (*matR*). By contrast, the mitochondrial genome of the Ogura CMS line possess 30 known genes, 69 ORFs, 3 rRNA genes and 25 tRNA genes (Supplementary Table 2). Interestingly, *rps7* was lost in the Ogura CMS mitochondrial genome.

### Collinearity analysis of the mitochondrial genomes

**Fig. 3:**
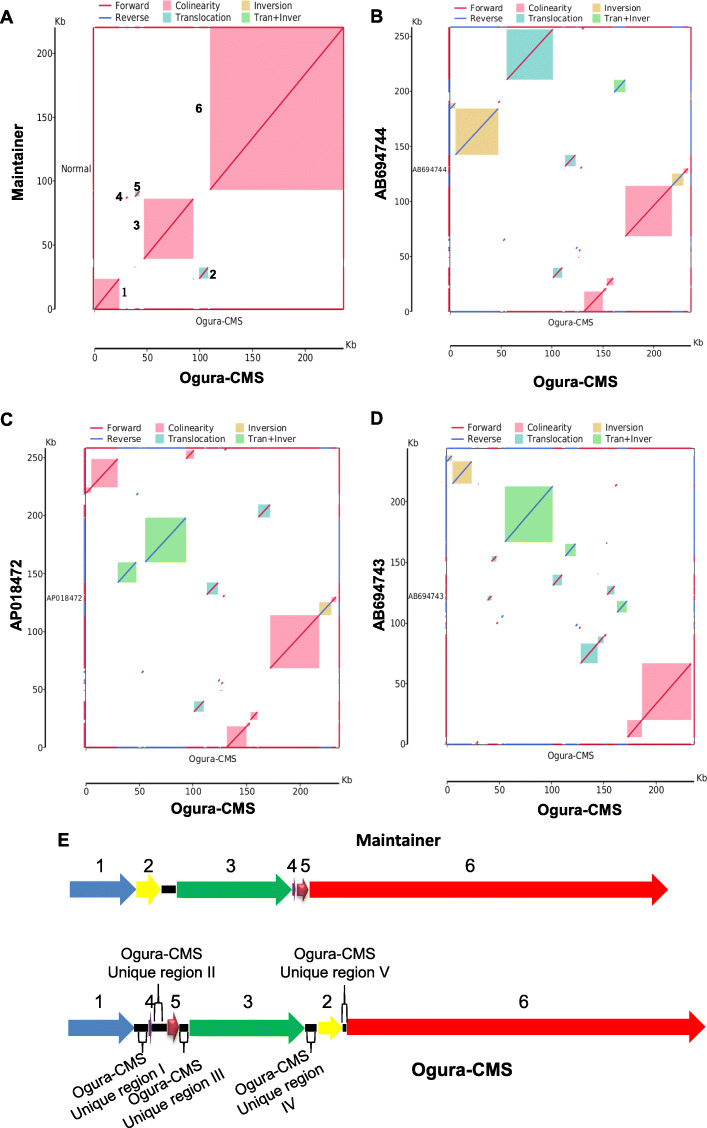
Collinearity analysis of the mitochondrial genomes. (**A**) Ogura CMS line genome on the X-axis, plotted against maintainer line genome on the Y-axis. Red lines indicate forward alignment, blue lines indicate reverse alignment. The numbers behind the inner squares for the syntenic regions correspond to those indicated in panel **A**. The color of the inner bars represent the alignment types; red color: Collinear; green color: Translocation; yellow color: Inversion. (**B**) to (**D**) Ogura CMS linear genome on the X-axis, plotted against the radish Ogura CMS cultivars MS-Gensuke (AB694744), Kosena (AP018472), Uchiki-gensuke (AB694743 ) genomes on the Y-axis. (**E**) Schematic illustration of six syntenic regions in the mitochondrial genomes of the maintainer and Ogura CMS cabbage lines. Six syntenic regions were named as block 1 to block 6. Ogura CMS had five unique regions

The maintainer and Ogura CMS cabbage mitochondrial genome, together with three published radish Ogura CMS mitochondrial genomes, were subjected to synteny analysis. The collinearity, inversion, translocation and Tran + Inver syntenic blocks were identified in each comparison (Fig. 3 A to 3D). The syntenic regions shared high sequence similarity between the maintainer and Ogura CMS cabbage mitochondrial genomes. However, the mitochondrial genomes of the Ogura CMS cabbage lines were separated into six syntenic regions by unique regions (Fig. 3 A and 3E). The collinearity analysis between Ogura CMS cabbage lines and different radish Ogura CMS lines showed that the unique regions are more likely to have come from the mitochondrial genome of the radish cultivar MS-Gensuke (AB694744) or cultivar Kosena (AP018472) than from cultivar Uchiki-gensuke (AB694743) (Fig. 3B and D). From the details of the collinearity analysis between the maintainer and Ogura CMS cabbage lines, we found six main syntenic regions (named as block 1 to block 6) (Fig. 3E), ranging from 1174 bp to 126,969 bp, which accounted for 89.71 and 96.52 % of the mitochondrial genome sequence in the Ogura CMS and maintainer lines, respectively. The corresponding blocks of the maintainer and Ogura CMS lines had at least 99 % identity. Block 1/block 4, block 4/block 5, block 5/block 3, block 5/block 3 and block 2/block 6 in the C5 CMS line are broken by unique region I, unique region II, unique region III, unique region IV and unique region V, respectively (Fig. 3E), which means that these regions had undergone recombination during the breeding process. Compared with the maintainer line, the Ogura CMS had three big forward syntenic regions (block 1, block 3 and block 6) and three small translocation syntenic regions (block 2, block 4 and block 5) (Fig. 3E). Our data showed that there had been an extensive recombination and rearrangement in the Ogura CMS cabbage mitochondrial genomes (Fig. 3).

### Analysis of the unique regions in the Ogura CMS mitochondrial genomes

**Fig. 4:**
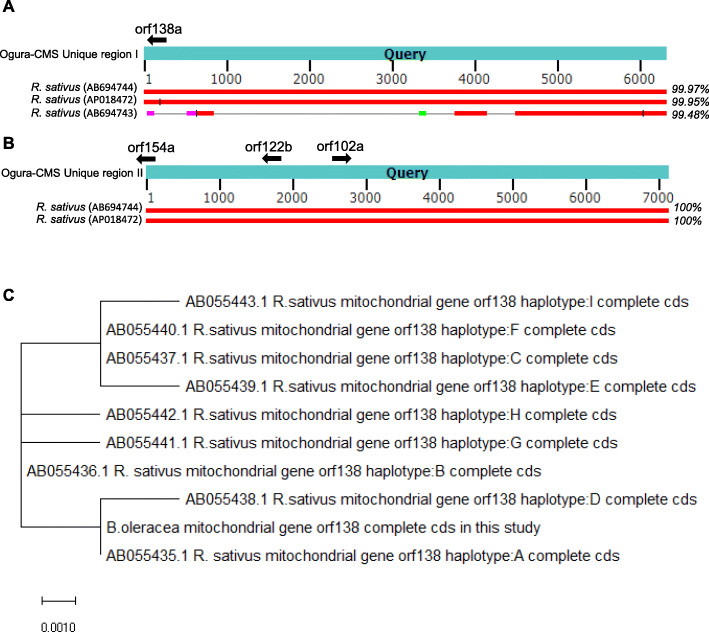
Ogura CMS Unique regions homologous to mitochondrial genomes of and evolutionary analysis of Ogura CMS orf138. Alignment of the Ogura CMS Unique region I (**A**) and Ogura CMS Unique region II (**B**) to the mitochondrial genomes of the maintainer line, *Raphanus sativus* (Ogura-type) and *Raphanus sativus* (normal-type). The query sequences are the Ogura CMS Unique region I (6291 bp) and Unique region II (7056 bp). orf138a is located at the edge of Unique region (I) orf154a is at the edge of Unique region (II) Different colored boxes indicate the sequence alignment scores to the three mitochondrial genomes. (**C**) Evolutionary analysis of Ogura CMS cabbage *orf138* with nine haplotypes from radish using the Maximum Likelihood method. The tree was drawn to scale, with branch lengths measured as the number of substitutions per site. The accession numbers of the orf138 sequences are shown in the tree

From the collinearity analysis, we found that the Ogura CMS mitochondrial genome has five unique regions that are non-homologous to the maintainer mitochondrial genome (Fig. 3 A). The size of unique regions I to V are 6291 bp, 7056 bp, 4225 bp, 5457 and 1715 bp, respectively. Total length of the unique regions is 25,125 bp, which accounts for 10.6 % of the whole Ogura CMS mitochondrial genome. BLASTN searching of nucleotide databases using the unique regions from I to V showed that the unique sequences have high similarity with mitochondrial sequences of radish cultivar MS-Gensuke (AB694744) or cultivar Kosena (AP018472). Ogura CMS unique region I has higher similarity with sequence of AB694744 (99.97 %) than that of AP018472 (99.95 %) (Fig. 4 A and 4B). The unique regions I to V were located at position 159,703 bp-165,993 bp, 151,092 bp-158,147 bp, 142,688 bp-146,912 bp, 249,243 bp-254,699 bp and 37,956 bp-39,670 bp of mitochondrial sequences of MS-Gensuke, respectively. Specific ORFs only appeared in unique regions I and II. *orf102a* and *orf122b* were located in the Ogura CMS unique region II, and *orf138a* was located at the left edge of the Ogura CMS unique region I (Table 1; Fig. 4 A); however, *orf154a* was located between Ogura CMS block 4 and unique region II (Table 1; Fig. 4B). The *orf138* sequences of Ogura CMS were classified into nine haplotypes from A to I [24]. We found that *orf138a* in our Ogura CMS cabbage line belongs to the haplotype A by nucleotide alignment and evolutionary analysis (Supplementary Fig. 2 and Fig. 4 C). Haplotype A sequence only appears in radish cultivar ‘MS-Gensuke’. Taken together, the original cytoplasm donor cabbage material was most likely generated by recombination with Ogura-type radish cultivar ‘MS-Gensuke’ through intergeneric hybridizations or cell fusion during the breeding process.

**Table 1:** The specific open reading frames (ORFs) in the Ogura CMS mitochondrial genome

Specific open-reading frames (orf)	the most similar mitochondrial sequence of other species	location in the Ogura CMS line
orf102a	YP_717109.1 hypothetical protein BrnapMp010 [Brassica napus]	In the Ogura-CMS Unique region II
orf122b	AEX57663.1 hypothetical protein RasatMp032 [Raphanus sativus]	In the Ogura-CMS Unique region II
orf138a	YP_006665998.1 hypothetical protein [Raphanus sativus]	In the Ogura-CMS Unique region I
orf154a	AEX57662.1 hypothetical protein RasatMp031 [Raphanus sativus]	Between Ogura-CMS block 4 and Unique region II

Four ORFs, including ORF102a, ORF122b, ORF138a, and ORF154a, were specifically identified in the Ogura CMS mitochondrial genome. ORF102a is closest to the mitochondrial protein of *Brassica napus*. ORF122b, ORF138a and ORF154a were most similar to the mitochondrial sequence of *Raphanus sativus*. ORF102a and ORF122b were located in the Ogura CMS unique region II and ORF138a was located at the left edge of the Ogura CMS unique region I; however, ORF154a was located between Ogura CMS block 4 and unique region II

### Identification of CMS-associated ORFs in the Ogura CMS mitochondrial genomes

**Fig. 5:**
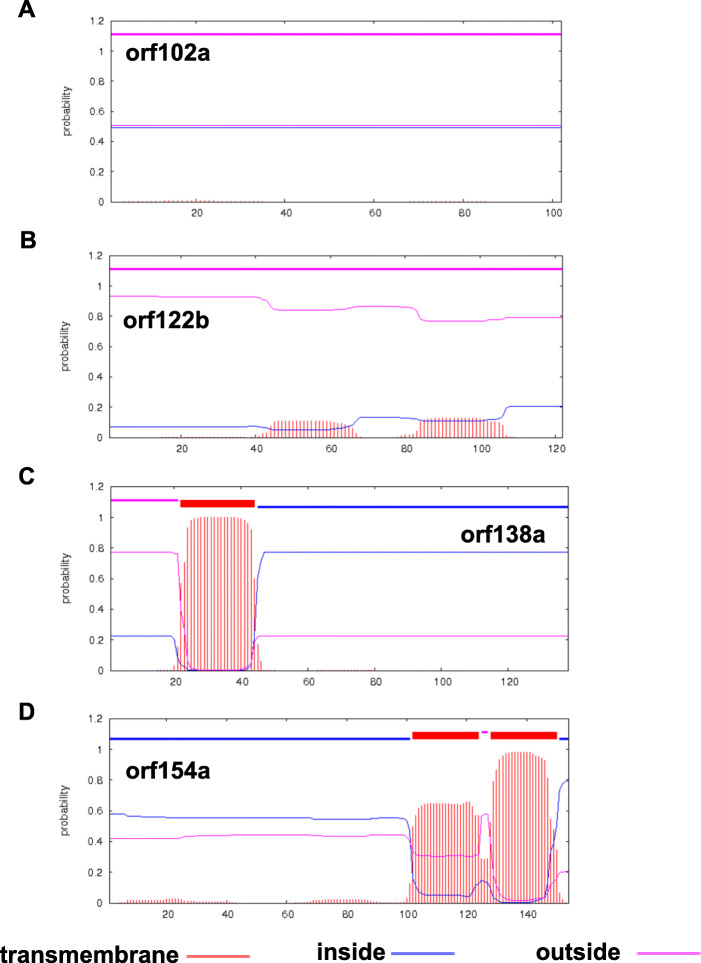
Transmembrane domain prediction of candidate CMS genes encoded proteins in the Ogura CMS line. The output of the TMHMM server shows the location and probability associated with the predicted transmembrane domains in the Ogura CMS line, **(A)** ORF102a, **(B)** ORF122b, **(C)** ORF138a, and **(D)** ORF154a

**Fig. 6:**
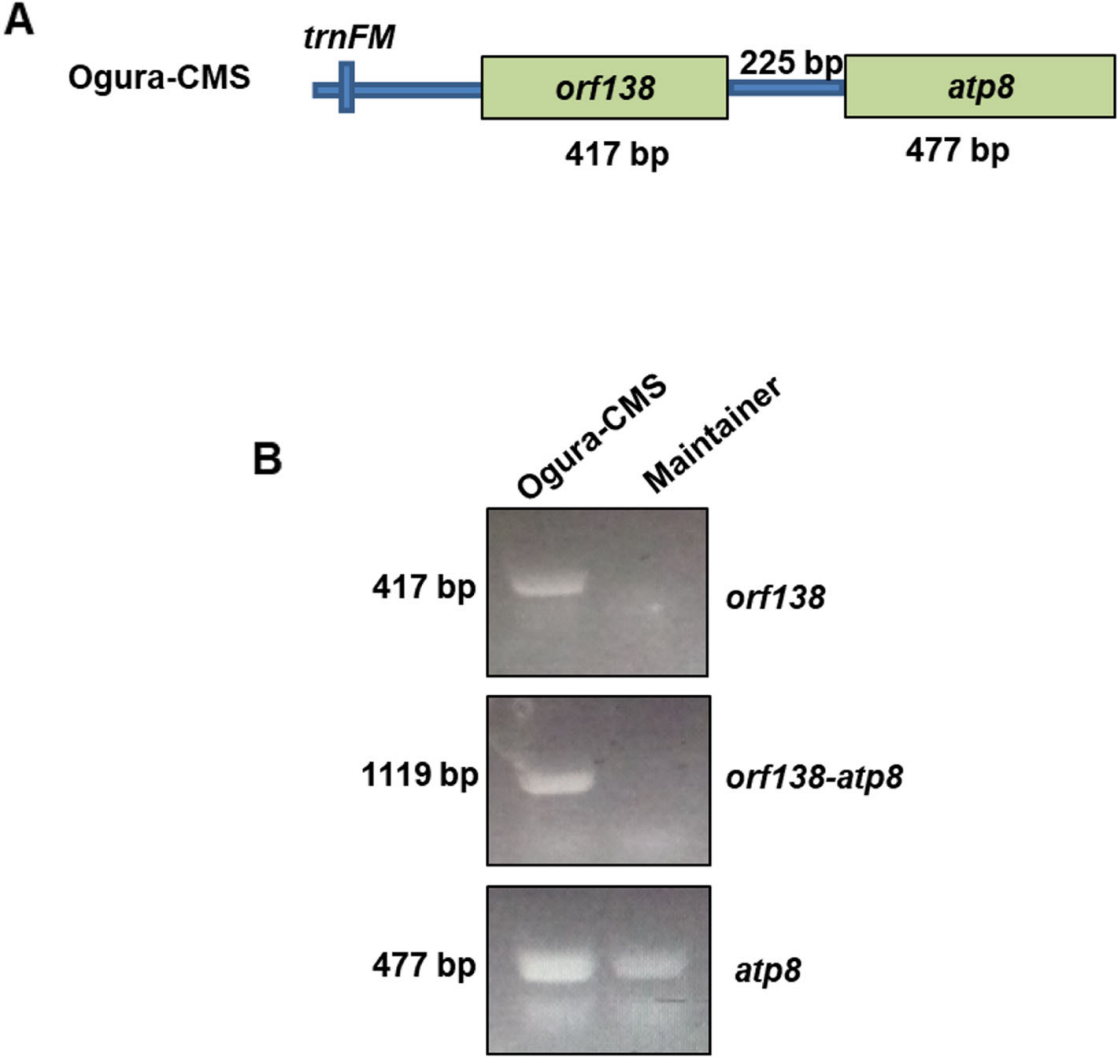
Co-transcription of *orf138a* and *atp8* in the Ogura CMS line. **(A) **Co-transcription structure of *orf138a *and *atp8*. **(B)**Transcription of *orf138a*/*atp8 *and co-transcription detection of *orf138a *and *atp8* were detected in the maintainer and Ogura CMS lines. The cDNA of Ogura CMS and maintainer lines derive from the same experiment and the gels were processed in parallel.The full-length gel is provided in Supplementary Figure 1

To reveal the genes determining the CMS phenomenon, we compared the mitochondrial genomes between the maintainer and Ogura CMS lines. Four ORFs that encoded over 100 amino acids, including ORF102a, ORF122b, ORF138a and ORF154a, were specifically identified in the Ogura CMS mitochondrial genome (Table 1). These specific genes in the unique region were generated by recombination, which is generally considered to control the CMS trait. We also found that ORF122b, ORF138a and ORF154a were identical to mitochondrial proteins of radish cultivar MS-Gensuke. To further verify whether these ORFs were candidate proteins for CMS, the structure of these three proteins were predicted. We found that ORF138a has only one transmembrane domain ; however, ORF154a possesses two transmembrane domains at the C-terminus of the protein, whereas ORF102a and ORF122b lack transmembrane domains (Fig. 5). In addition, *orf154a* was located between Ogura CMS block 4 and Unique region II of mitochondrial genome of Ogura CMS lines. *orf154a* has partial homologous sequences to the ATP synthase subunit 1 (*atpA*) gene. *orf138a* is co-transcribed with *atp8*, which was confirmed using reverse transcription polymerase chain reaction (RT-PCR) (Fig. 6). In conclusion, our data indicated that both *orf138a* and *orf154a* were likely responsible for Ogura CMS.

### Detection of ATP production in the Ogura CMS cabbage lines

**Fig. 7:**
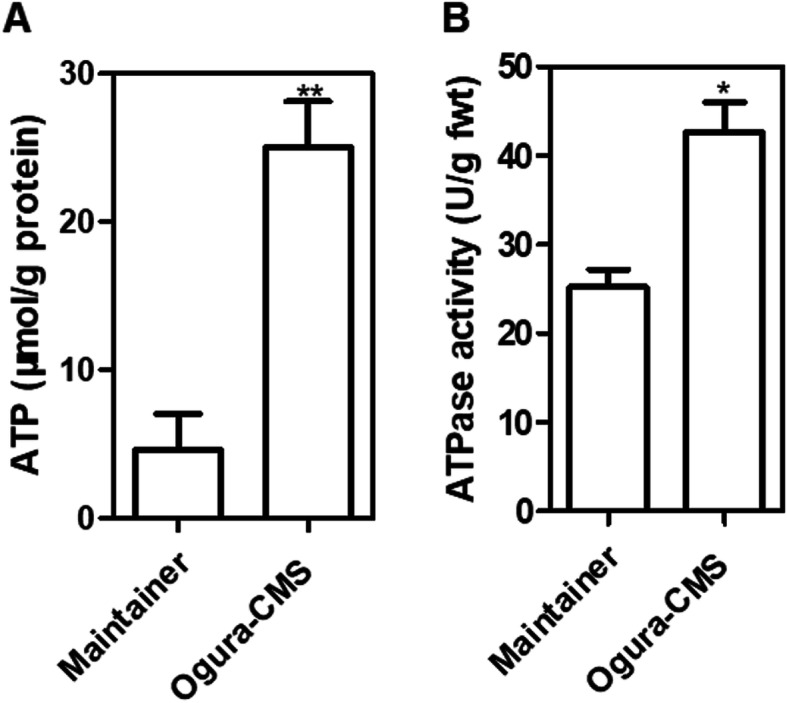
The production of ATP and ATP synthase activity in petal samples of maintainer and Ogura CMS cabbage lines. **(A)** Analyses of the ATP content in petal of maintainer and Ogura CMS cabbage lines. **(B)** Analyses of the ATP synthase activity in petal of maintainer and Ogura CMS cabbage lines. Shown are t-test comparison statistics (means ± SD; *n* = 3). *P < 0.05; **P < 0.01; ***P < 0.001

From our whole mitochondrial genome data, we found that the *orf138a* was co-transcribed with atp8, and *orf154a* has partial homology to the *atpA* gene; therefore, we wondered whether the functions of *orf138a* and *orf154a* are associated with the yield of ATP. To answer this question, we measured the ATP content in anther samples from maintainer and Ogura CMS cabbage lines. We found that the ATP level in the anther samples of Ogura CMS cabbage lines was remarkably higher than that in the maintainer lines (Fig. 7 A). The change in ATP yield may cause by altered ATPase activity. To test this hypothesis, ATPase activity was detected in maintainer and Ogura CMS cabbage lines. As expected, slightly higher ATPase activity was detected in the anther samples of Ogura CMS cabbage lines compared with that in the maintainer lines (Fig. 7B). In conclusion, the alterations in ATPase activity and ATP content indicate that *orf138a* and *orf154a* might influence the mitochondrial energy metabolic pathways.

### Paraffin section analysis of anther development in the maintainer and Ogura CMS cabbage lines

**Fig. 8:**
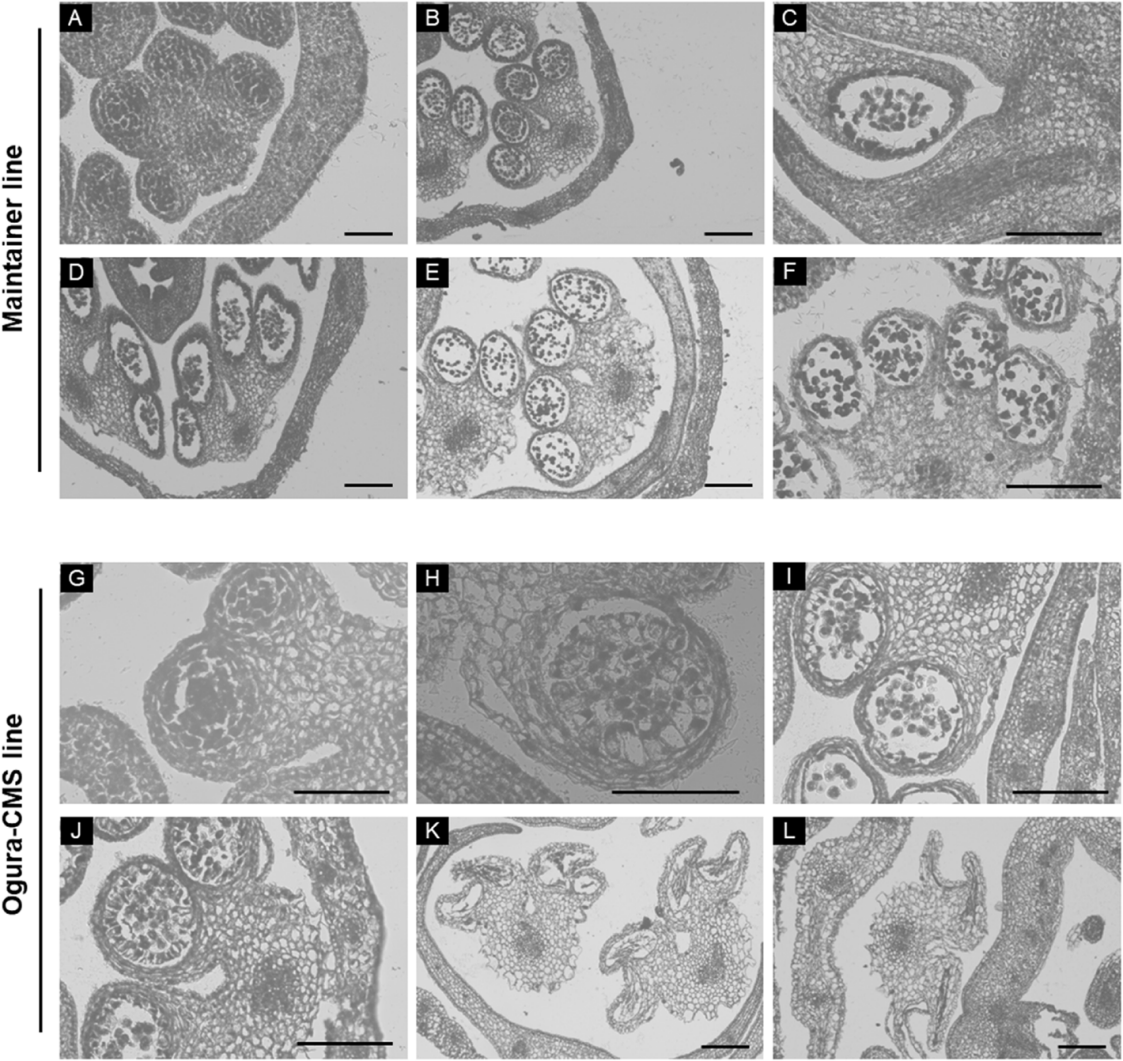
Cytology of anther development of maintainer and Ogura CMS cabbage lines. **(A)** and **(G)** show the sporogenesis cell stage; **(B)** and **(H)** show the microspore mother cell stage; **(C)** and **(I)** show the tetrad stage; **(D)** and **(J)** show the uninucleate microspore stage; **(E)** and **(K)** show the mature pollen stage; **(F)** and **(L)** show the dehiscence stage. Scale bar = 50 μm

From our mitochondrial sequence data, the cabbage Ogura CMS possesses the *orf138a* gene from radish, which is thought to be responsible for the male-sterile phenotype in most CMS systems. However, the vegetative growth of these two lines is not different. Meanwhile, the mechanism of male sterility gene on pollen abortion is still uncertain. Consequently, we performed comparative cytological analysis of anther development in the maintainer and Ogura CMS cabbage lines to demonstrate which cell types were affected in the stamens of the Ogura CMS line, and to further determine the cause of Ogura CMS of cabbage. In the maintainer line, the stamen meristem differentiates into the anther primordia with four microsporangiums; then the archesporial cells in each microsporangium differentiates into secondary parietal cell layers and sporogenous cells (Fig. 8 A). Then, the microspore mother cells, tapetum, middle layer, endothecium and epidermis are generated after asymmetric and symmetrical cell divisions (Fig. 8B). The tetrads containing microspores enclosed by the callose wall are formed after meiosis II (Fig. 8 C). Free haploid microspores are released from the tetrads during the uninucleate microspore stage (Fig. 8D). The mature pollen grain is then generated, and the tapetal cells begin to separate from the middle layer (Fig. 8E). At last stage, the tapetum completely disappears and anther dehiscence occurs with the release of mature pollen grains (Fig. 8 F). However, in the Ogura CMS line, everything seemed normal at sporogenesis cell stage (Fig. 8G), haploid microspores can be formed and released properly (Fig. 8I J); however, tapetal cells began to show abnormal activity at the end of the meiosis II stage, becoming thicker (Fig. 8 H, 8I and 8 J). Expanded tapetal cells hindered the development of haploid microspores through spatial oppression, which resulted in the structural disruption of haploid microspores. Finally, both haploid microspores and tapetal cells degenerate quickly (Fig. 8 K and 8 L). Taken together, the abnormal proliferation of tapetal cells might be the immediate cause of cytoplasmic male-sterility in Ogura CMS cabbage lines.

### Comparative RNA-seq-based transcriptional profiling of maintainer and Ogura CMS lines

**Fig. 9:**
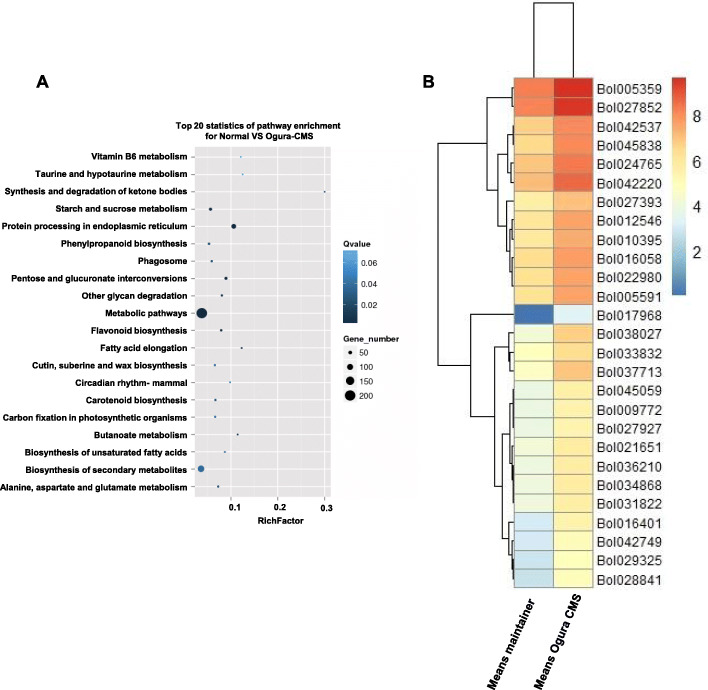
Presence of *orf138a* leads to major transcriptional changes of genes in metabolic pathways. **(A**) Statistics of the top 20 pathway classifications from GO enrichment analysis of genes differentially expressed in maintainer and Ogura CMS cabbage lines (based on RNA-seq analyses). The number of genes and Q-values are indicated by the size of circles and the color of the rectangles, respectively. (**B**) Heatmap showing that the relative expression of the 27 differentially expressed metabolic and biosynthesis of secondary metabolites-related genes based on the FPKM method

To further understand the molecular mechanisms underlying Ogura CMS, whole genome transcriptional profiling between the maintainer and Ogura CMS lines was investigated using RNA-seq analysis. According to the Gene Ontology (GO)enrichment analysis of differentially expressed genes (DEGs) between the two lines, we found that the DEGs were significantly enriched in starch and sucrose metabolism (ko00500), biosynthesis of secondary metabolites (ID: ko01040) and metabolic pathway (ID: ko01100) (Fig. 9 A). These results indicated that the presence of *orf138* impacts energy metabolism pathways. To identify the metabolic pathway in which the DEGs are involved, we performed Heatmap analysis based on the ‘fragments per kilobase of exon per million mapped reads’ (FPKM) method (Fig. 9B and Supplementary Table 4). Interestingly, the *Bol016401* gene encoding cytochrome bd ubiquinol oxidase was up-regulated in the Ogura CMS lines, which is involved in the production of ATP in the mitochondrial electron transport chain (ETC). In addition, *Bol036210* (SQP1, FAD/NAD (P)-binding oxidoreductase family protein) functions in the ETC, based on its activity as a monooxygenase. Furthermore, *Bol033832* (NAD (P)-linked oxidoreductase superfamily protein) and *Bol027393* (HSR8, NAD (P)-binding Rossmann-fold superfamily protein) participate in the ETC according to their oxidoreductase activities. The RNA-seq results showed that presence of *orf138a* and *orf154a* in the Ogura CMS might influence the transcript levels of genes in energy metabolic pathways.

## Discussion

### Original Ogura CMS cabbage mitochondrial genome was generated by recombination with Ogura type radish

There are three origins of Brassicaceae CMS, including intraspecific variations, alloplasmic origin by interspecific or intergeneric hybridizations, and cell fusion [25]. The *pol* CMS, 681 A CMS of *B. napus* and *hau* CMS in *B. juncea* are three examples of spontaneous male sterility [8, 26, 27], which were caused by intraspecific variations. Some CMS lines in *B. oleracea* were obtained by sexual hybridization with *B. rapa*, *B. nigra*, *B. napus*, *R. sativus (Early scarlet Globe)* and *R. sativus* (*Ogura*) [4]. *R. sativus* (*Ogura*) was usually used to produce CMS plants by cell fusion, generating the CMS lines with abortion in the tetrad stage [28]. In the present study, the complete mitochondrial genomes of maintainer and Ogura CMS cabbage were constructed through sequencing and de novo assembly. Syntenic regions analysis of the mitochondrial genomes between these two cabbage lines showed that the Ogura CMS cabbage mitochondrial genome has five unique regions (Fig. 3 A). These regions are non-homologous to maintainer cabbage mitochondrial genome, but are identical to the sequence reported for radish Ogura-type mitochondrial genomes (AB694744) (Fig. 4). In conclusion, the original cytoplasm donor cabbage material was recombined from normal cabbage and Ogura CMS radish (MS-Gensuke) by intergeneric hybridizations or cell fusion.

### Candidate genes controlling the Ogura CMS of cabbage

Candidate genes were selected for the CMS-associated genes based on the following characteristics: Novel chimeric structure, transmembrane domains and co-transcription events. For instance, *orf138, orf300a, orf463, orf222* and *orf224* have been confirmed to be generated by recombination of mitochondrial genome, possessing above mentioned characters, and are responsible for the Ogura CMS in Brassica [9], CMS in pepper [29], Dongbu cytoplasmic and genic male-sterility (DCGMS) CMS in radish [28], and *nap* and *pol* CMS in *Brassica napus* [1, 11], respectively. In the present study, ORF102a, ORF122b, ORF138a and ORF154a were specific ORFs in the Ogura CMS cabbage mitochondria, but only ORF138a and ORF154a were transmembrane proteins. *orf154a* has partial homologous sequences to the ATP synthase subunit 1 (*atpA*) gene. Furthermore, *orf138a* is co-transcribed with atp8 (Fig. 6). According to the energy deficiency model, CMS-associated ORF genes might disturb ATP synthesis [14]. For instance, ORFH79 can impair ATP production through binding to complex III and decreasing its enzyme activity, which results in abnormal pollen development in Honglian CMS rice [30]. The CMS associated gene *orf188* promotes ATP production during mitochondrial oxidative phosphorylation [31]. Here, we found that the ATPase activity and ATP content were higher in the anther samples of the Ogura CMS cabbage line (Fig. 7), which means that *orf138a* and *orf154a* might affect the mitochondrial energy metabolic pathways. It was reported that alterations of mitochondria-encoded subunits of the F_0_F_1_-ATP synthase triggered CMS in plants because of the high ATP demand of floral tissues [32, 33]. Furthermore, our RNA-seq results confirmed that *orf138a* and *orf154a* in the Ogura CMS might affect the transcript levels of genes in energy metabolic pathways, especially those in the ETC (Fig. 9). Moreover, *rps7* was lost in Ogura CMS cabbage because of the Unique region II insertion from radish. In a few cases, any disruption of the electron transport chain could impair energy production [34, 35]. Taken together, the disordered energy production in mitochondria triggers male sterility, which supports the mitochondrial energy deficiency model. In addition, *orf138* is usually considered as the main candidate gene for the Ogura CMS. Actually, four genes *orf102a*, *orf122b*, *orf138a*, and *orf154a*, from other species were identified in our whole mitochondrial genome date. More functional analysis of *orf138a* and *orf154a* are required to understand the sterility mechanism in Ogura CMS of cabbage.

### Abnormal proliferation of tapetal cells probably causes male sterility in Ogura CMS cabbage

The abortive stages of anther development in many CMSs of Brassica plants have been analyzed by cytological analysis. For example, premature cell death events of the tapetal cells impairs pollen development at the vacuolate microspore stage, leading to male sterility in Ogu-INRA CMS of rapeseed (*Brassica napus*) [36]. The abortion of anther development in the recessive male sterility cabbage (83,121 A) is caused by a lack of sporopollenin deposition and exine formation [37]. The dominant male sterility gene *Ms-cd1* was found to suppress the expression of certain genes in tapetal cells, which might prevent the degradation of callose and pollen mother cell (PMC) wall in *B. oleracea* [38]. Our cytological sections data showed that the abnormal proliferation of tapetal cells hindered the development of haploid microspores through spatial constriction, which resulted in the structural disruption of haploid microspores in the Ogura CMS cabbage lines (Fig. 8 K and 8 L). The RNA-seq results showed the transcription of genes in energy metabolic pathways was activated (Fig. 9), and the ATPase activity and ATP content were increased in the Ogura CMS lines (Fig. 7), which could provide the more energy for the abnormal proliferation of tapetal cells. Taken together, tapetal cells play an important role in the anther development. The abnormal PCD of tapetal cells might be the immediate cause of cytoplasmic male-sterility in our Ogura CMS cabbage lines.

## Conclusions

We found that Ogura CMS cabbage lines consist of six syntenic regions from collinearity analysis of the mitochondrial genomes. ORF122b, ORF138a and ORF154a were highly similar to mitochondrial proteins of *Raphanus sativus*. Thus, our cabbage Ogura CMS line was probably generated through intergeneric hybridizations or cell fusion between cabbage and radish. Among these ORFs, *orf138a* and *orf154a* possessed the transmembrane structure, and *orf138a* was co-transcribed with atp8 and *trnfM, orf154a* has partial homologous sequences to the *atpA* gene. The ATPase activity and ATP content were higher in the in anther samples of Ogura CMS cabbage lines, suggesting that *orf138a* and *orf154a* affect ATP production in the mitochondrial energy metabolic pathways. The RNA-seq data also confirmed that the transcript levels of genes in the metabolic pathways were increased in the Ogura CMS lines. In addition, cytological sections showed that the abnormal proliferation of tapetal cells might be the immediate cause of cytoplasmic male-sterility in Ogura CMS cabbage lines. Taken together, *orf138a* and *orf154a* lead to increased ATPase activity and ATP content by affecting the transcriptional levels of genes in energy metabolic pathways, which could provide the more energy for the abnormal proliferation of tapetal cells.

## Methods

### Plant materials

R2P2 is an open-pollination and early maturing cabbage variety. The original cytoplasm donor cabbage material was introduced from Germany in 2004 by Dr. Yuancai Jian from the Beijing Vegetable Research Center of Beijing Academy of Agriculture and Forestry Sciences (BAAFS). It was confirmed as a male sterile material with the Ogura cytoplasm containing *orf138*. R2P2 was crossed with donor cabbage material and backcrossed for eight generations to generate the BC8 CMS cabbage line R2P2CMS, with R2P2 as the paternal line in all crosses. The stability of R2P2CMS was observed for more than 10 years in two experimental fields of the Beijing Vegetable Research Center (BVRC). The mitochondrial DNA of Ogura CMS (R2P2CMS) and maintainer (R2P2) lines were sent to Biozeron Company (Shanghai, China) for DNA library construction and sequencing. R2P2CMS and R2P2 lines were grown under the same conditions. After flowering, six inflorescences (with the same size) from several CMS plants and six inflorescences from several maintainer plants were harvested, respectively. In each line, six inflorescences were further randomly assigned to two subgroups. These four subgroup samples, named R2P2CMS-1, R2P2CMS-2, R2P2-1, and R2P2-2, were stored in liquid nitrogen and then at -80 °C for RNA-seq analysis. 

### Mitochondrial genomes sequencing and assembling

About 5 g of fresh cabbage leaves was harvested for mtDNA isolation using a modified extraction method [39]. One microgram of purified mtDNA was fragmented to generate 430 bp short-insert libraries following the manufacturer’s instructions (Illumina, San Diego, CA, USA), and sequenced on the Illumina Hiseq 4000 platform [40]. The high molecular weight DNA was purified for PacBio library prep, BluePippin size selection, and sequenced on the Pacbio Sequel Sequencer (PacBio Inc., Menlo Park, CA, USA). Then, the adapters, reads containing over 10 % Ns (uncalled bases), duplicated sequences and low-quality reads (the Phred scores < Q20) were removed. The Phred scores (Q20, Q30) and GC content of the clean data were calculated. The mitochondria genome framework was constructed using both Illumina Hiseq and Pacbio Sequel data using ABySS v2.0.2 and SPAdes v3.10.1 software [41]. The contig gaps were filled to complete the circular or linear mitochondria genomes of maintainer and Ogura CMS cabbage lines. The circular genome maps were drawn using OrganellarGenomeDRAW v1.2 [42].

### Reverse transcription-PCR

Total RNA was isolated from the anthers of maintainer and Ogura CMS lines using TRIzol® Reagent (Invitrogen Waltham, MA, USA), and first-strand cDNA was synthesized from total RNA using a reverse transcriptase (Takara, Dalian, China). RT-PCR amplification was performed with the following thermal cycles: An initial denaturation step at 94 °C for 5 min; 35 cycles of 94 °C for 10 s, 55 °C for 1 min, and 72 °C for 1 min; and a final 10-min extension at 72 °C. Primers for the RT-PCR assays are shown in Supplementary Table 3.

### Genome annotation

The mitochondria genes were annotated using combination of homology alignment and de novo prediction. Opening reading frames (ORFs) in the mitochondrial genome were identified using ORF Finder (http://www.ncbi.nlm.nih.gov/gorf/gorf.html). Ribosome RNA (rRNA) and transfer RNA (tRNA) genes were predicted using rRNAmmer 1.2 and tRNAscan-SE, respectively [43, 44]. A whole mitochondria genome blast search was performed against the following databases: KEGG (Kyoto Encyclopedia of Genes and Genomes) [45], COG (Clusters of Orthologous Groups) [46], NR (Non-Redundant Protein Database database), Swiss-Prot [47], and GO (Gene Ontology) [48]. Transmembrane domains in each candidate ORF were assessed using TMHMM Server v.2.0 (http://www.cbs.dtu.dk/services/TMHMM/).

### ATP content and synthase activity measurement

The anthers of maintainer and Ogura CMS lines were harvested for ATP content and synthase activity quantification. The ATP content and synthase activity were measured using an ATP content assay kit and an Na^+^K^+^-ATP synthase activity assay kit by detecting luciferase signals using a multifunctional microplate reader at 340 nm and 660nm, respectively (Solarbio Co., Ltd., Beijing, China). The detailed procedures were carried out according to the manufacturer’s instructions ((Solarbio, BC0300 and BC0065).

### Cytological analysis

Preparation and observation of ultra-thin sections: The anthers of maintainer and Ogura CMS lines were fixed in formalin-aceto-alcohol (FAA) solution. The samples were dehydrated with a graded ethanol series (30, 50, 70, 80, 90, 95, and 100 %) after rinsing with distilled water. The anthers were embedded in Spur (ERL-4206) and sectioned into 3 mm^2^ sections (approximately 2000 nm thick) using a Leica ultracut R ultramicrotome (Leica Wetzlar, Germany). The samples were dried overnight in a 70 °C incubator, and then stained with 1 % toluidine blue or 0.5 % crystal violet stain solution. A Leica DMR2 microscope was used to observe the cell structure of anther; the images were photographed using a Nikon Coolpix4200 camera (Nikon, Tokyo, Japan). Preparation and observation of sample for transmission electron microscopy (TEM): Fresh anthers of maintainer and Ogura CMS lines were fixed overnight in 2 % glutaraldehyde. The samples were dehydrated with a graded ethanol series (30, 50, 70, 80, 90, 95, and 100 %) after rinsing with distilled water. The anthers were embedded in epoxy resin 618 (Bluestar Wuxi Petrochemical Co., Ltd, China) and sectioned into 3 mm^2^ sections (approximately 2000 nm thick) using a Leica ultracut R ultramicrotome. The samples were stained with uranyl acetate and lead nitrate solution. The images were photographed using a Hitachi H-7500 transmission electron microscope (Hitachi, Tokyo, Japan).

### Illumina sequencing and transcriptome analysis

Total RNA was extracted from the inflorescence of Ogura CMS and its maintainer line using an RNAprep Pure Plant Kit (Tiangen Co., Ltd., Beijing, China) following the manufacturer’s protocol. High-quality RNA from each sample was used for cDNA library construction and RNA-Seq on the Illumina HiSeqTM 2000 platform at the Beijing Genomics Institute (BGI, Beijing, China). The clean reads were obtained by filtering the adaptor reads, low quality reads (> 50 % bases had Q-values ≤ 5) and unidentified bases (“N” reads > 10 %). The clean reads were mapped to the *B. oleracea* genome using the TopHat2 package (2.0.10) [49]. Gene Ontology (GO) classification for understanding the distribution of gene function was conducted by using WEGO software [50]. Pathways with Q-value < 0.05 were considered as significantly enriched for the DEGs. The abundance of differentially expressed transcripts related to metabolism and biosynthesis of secondary metabolite was estimated by calculating read density as ‘fragments per kilobase of exon per million mapped reads’ (FPKM) using Cuffdiff (v2.1.1) [51].

## Supplementary Information

**Additional file 1.**

**Additional file 2.**

**Additional file 3.**

**Additional file 4.**

**Additional file 5.**

**Additional file 6.**

## Data Availability

The datasets supporting the conclusions of this article are available in the GenBank nucleotide sequence database repository [MW423604 and MW423605; https://www.ncbi.nlm.nih.gov/genbank/].

## Abbreviations

CMS: Cytoplasmic male sterility
mtDNA: Mitochondrial DNA
ORF: Open reading frame
ORF: Open reading frame
ATP1, ATP4, ATP6, ATP8, and ATP9: ATP synthase subunits 1, 4, 6, 8, and 9 genes
COB: Apocytochrome b gene
COX1-3: Cytochrome c oxidase subunits 1–3 genes
NAD1-7, 9 and NAD4L: NADH dehydrogenase subunits 1–7, 9, and 4 L genes
RPL2, RPL5, RPL16, RPS4, RPS7, RPS12 and RPS14: ribosomal protein large subunit genes
tRNA: Transfer RNA (tRNA) genes
