# Supplementary material for: Comparative analysis of the complete mitochondrial genome sequences and anther development cytology between maintainer and Ogura-type cytoplasm male-sterile cabbage (B. oleracea Var. capitata)
Source: BMC Genomics. 2021 Sep 7;22:646. doi: 10.1186/s12864-021-07963-x (PMC8425178; doi:10.1186/s12864-021-07963-x)
Supplement: Supplementary file 2 — Additional file 2. [file 12864_2021_7963_MOESM2_ESM.docx]

**Comparative analysis of the complete mitochondrial genome sequences and anther development cytology between maintainer and Ogura-type cytoplasm male-sterile cabbage (*B. oleracea* Var. *capitata*)**

Xionghui Zhong^1^, Denghui Chen^1, 2^, Jian Cui^1^, Hailong Li^1^, Yuxin Huang^1^, Jungen Kang^1,^*

^*^Correspondence: [kangjungen@nercv.org](mailto:kangjungen@nercv.org);

^1^ Beijing Vegetable Research Center, Beijing Academy of Agriculture and Forestry Sciences, Key Laboratory of Biology and Genetic Improvement of Horticultural Crops (North China), Ministry of Agriculture, Beijing 100097, P.R. China

^2^ College of Horticulture, Gansu Agricultural University, Lanzhou 730070, P.R. China;

**Supplementary Fig. 2 Nucleotide alignment of Ogura CMS cabbage line *orf138* sequence and nine Haplotypes of *orf138* sequences from radish. Haplotypes A (AB055435), Haplotypes B (AB055436), Haplotypes C (AB055437), Haplotypes D (AB055438), Haplotypes E (AB055439), Haplotypes F (AB055440), Haplotypes G(AB055441), Haplotypes H (AB055442) and Haplotypes I (AB055443).**
